# Interspecific competition between entomopathogenic nematodes (*Steinernema*) is modified by their bacterial symbionts (*Xenorhabdus*)

**DOI:** 10.1186/1471-2148-6-68

**Published:** 2006-09-05

**Authors:** Mathieu Sicard, Julie Hinsinger, Nathalie Le Brun, Sylvie Pages, Noël Boemare, Catherine Moulia

**Affiliations:** 1 Laboratoire Génome, Populations, Interactions, Adaptation UMR 5171 CNRS, Université de Montpellier 2, Place Eugène Bataillon cc. 63, 34095 Montpellier, France; 2 Laboratoire de Génétique et Biologie des Populations de Crustacés, UMR 6556 CNRS, Université de Poitiers, 40 avenue du Recteur Pineau, 86022 Poitiers, France; 3 Laboratoire Ecologie microbienne des insectes et interactions hôte-pathogène UMR 1133 INRA, Université de Montpellier 2 cc. 54, 34095 Montpellier, France

## Abstract

**Background:**

Symbioses between invertebrates and prokaryotes are biological systems of particular interest in order to study the evolution of mutualism. The symbioses between the entomopathogenic nematodes *Steinernema *and their bacterial symbiont *Xenorhabdus *are very tractable model systems. Previous studies demonstrated (i) a highly specialized relationship between each strain of nematodes and its naturally associated bacterial strain and (ii) that mutualism plays a role in several important life history traits of each partner such as access to insect host resources, dispersal and protection against various biotic and abiotic factors. The goal of the present study was to address the question of the impact of *Xenorhabdus *symbionts on the progression and outcome of interspecific competition between individuals belonging to different *Steinernema *species. For this, we monitored experimental interspecific competition between (i) two nematode species: *S. carpocapsae *and *S. scapterisci *and (ii) their respective symbionts: *X. nematophila *and *X. innexi *within an experimental insect-host (*Galleria mellonella*). Three conditions of competition between nematodes were tested: (i) infection of insects with aposymbiotic IJs (i.e. without symbiont) of both species (ii) infection of insects with aposymbiotic IJs of both species in presence of variable proportion of their two *Xenorhabdus *symbionts and (iii) infection of insects with symbiotic IJs (i.e. naturally associated with their symbionts) of both species.

**Results:**

We found that both the progression and the outcome of interspecific competition between entomopathogenic nematodes were influenced by their bacterial symbionts. Thus, the results obtained with aposymbiotic nematodes were totally opposite to those obtained with symbiotic nematodes. Moreover, the experimental introduction of different ratios of *Xenorhabdus *symbionts in the insect-host during competition between *Steinernema *modified the proportion of each species in the adults and in the global offspring.

**Conclusion:**

We showed that *Xenorhabdus *symbionts modified the competition between their *Steinernema *associates. This suggests that *Xenorhabdus *not only provides *Steinernema *with access to food sources but also furnishes new abilities to deal with biotic parameters such as competitors.

## Background

Symbioses between the entomopathogenic nematodes *Steinernema *spp. and the enterobacteriacae *Xenorhabdus *spp. are associations in which both partners receive benefits from each other [1-3]. In the soil, the infective juveniles (IJs) of the nematodes act as vectors dispersing the bacteria from insect host to insect-host and in turn, the bacteria increase the nematode's fitness within the insects hosts [3,4]. Previous studies showed that these symbioses were highly specific and that no *Steinernema *spp. was able to associate with a *Xenorhabdus *spp. genetically distant from its natural one [2,5,6]. As the bacterial dispersion is totally dependent upon the fitness of the nematode within the insect-host, it is possible that *Xenorhabdus *spp. might select special traits in order to enhance their vector's fitness. It is known that *Xenorhabdus *spp. are beneficial to their nematodes in providing the latter with a better ability to kill the insect and feed on it [1,7,8]. Previous studies that focused on two different *Steinernema *species (*S. carpocapsae *and *S. scapterisci*) have provided us with insights into the association characteristics [2,5,6,9-11]. Although the two nematode species demonstrated increased fitness when they parasitized insect-hosts with their own native symbiont, *S. scapterisci *appeared less dependent upon its native symbiont (*X. innexi *[12]) than *S. carpocapsae *(associated with *X. nematophila*). Thus, *S. scapterisci*'s symbiont increased the reproductive rate of its naturally associated nematode by a factor of 1.3, whereas *X. nematophila *increased the reproductive rate of its naturally associated nematode by sevenfold (i.e. *S. carpocapsae*) [3]. Moreover, *S. scapterisci*, transported 700-fold fewer cells of its *Xenorhabdus *than *S. carpocapsae *(i.e. ~50 bacteria per nematode for *S. carpocapsae *and ~0.07 bacteria per nematode for *S. scapterisci*) [3]. These two *Steinernema *species also differed in their ability to deal with non-native *Xenorhabdus *strains in case of co-infection in an insect [5,6]. While *S. scapterisci *reproduced in co-infection situations with all the tested *Xenorhabdus *strains (even if its reproduction was better with its native one than with others), *S. carpocapsae *could not reproduce at all with most of them in the same situation [5,6]. Despite these specific differences, the global trend emerging from these previous experiments was that non-naturally associated *Xenorhabdus *strains tend to be antagonist against nematodes species which cannot disperse them. One can easily think that this antagonistic effect of *Xenorhabdus *strains on the fitness of nematodes naturally associated with others *Xenorhabdus *strains could be selected in case of frequent interspecific competition. In such an evolutionary context, we can postulate that each *Xenorhabdus *strain should try to provide its own nematode-vector with competitive advantages by producing antagonistic molecules against foreign nematodes. Indeed, a previous study has shown with the association *S. carpocapsae-X. nematophila *as a model-system that, in insects co-infected by antagonistic *Xenorhabdus*, *X. nematophila *partly counteracted their antagonistic effect on the nematodes fitness most probably by the mean of bacteriocins [13,14].

The goal of the present study was to address the question of the impact of *Xenorhabdus *symbionts on the progression and outcome of interspecific competition between individuals belonging to different *Steinernema *species. For this, we monitored experimental interspecific competition between (i) two nematode species: *S. carpocapsae *and *S. scapterisci *and (ii) their respective symbionts: *X. nematophila *and *X. innexi *within an insect-host (*Galleria mellonella*). In this study, three conditions of competition between nematodes were tested: (i) infection of insects with aposymbiotic IJs (i.e. without symbiont) of both species (ii) infection of insects with aposymbiotic IJs of both species in presence of variable proportion of their two *Xenorhabdus *symbionts and (iii) infection of insects with symbiotic IJs (i.e. naturally associated with their symbionts) of both species.

## Results

### Proportion of each *Xenorhabdus *in insect's hemolymph 72 h post-infection with IJs

**Figure 1:**
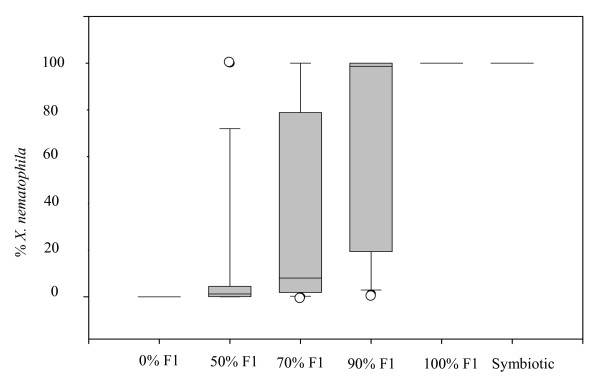
Percentile distribution of the percentages of *X. nematophila *within *Xenorhabdus sp*. found in the hemolymph of the insects 48 h post-infection. Results are given as box plots, where the horizontal line indicate the median (50^th ^of the data), the bottom and the top of the box indicate the first quartiles (25^th ^of the data) and the third quartiles (75^th ^of the data). The whiskers the range of the data (10^th ^of the data and 90^th ^of the data). Others dots are outliers which are under the 10^th ^percentile of the data or over 90^th ^percentile of the data. The abscissa shows the condition of the initial infection.

We observed that both bacterial strains were able to multiply and co-exist within the hemolymph of the insect. Nevertheless, *X. nematophila *was clearly less represented within the hemolymph 72 h post-infection with nematodes when an initial injection of a suspension containing 50% of each bacterium in the insect was performed (Fig 1). When 70% and 90% of *X. nematophila *were injected, the two bacteria were found in a very variable proportion and co-existed (Fig 1). In the competition resulting from infection of insects with symbiotic IJs of both nematode species, as well as when 100% of *X. nematophila *were injected into insects infected with aposymbiotic IJs of both nematode species, no *X. innexi *were detected within the hemolymph (Fig 1).

### Assessment of nematodes maturation in competition

**Figure 2:**
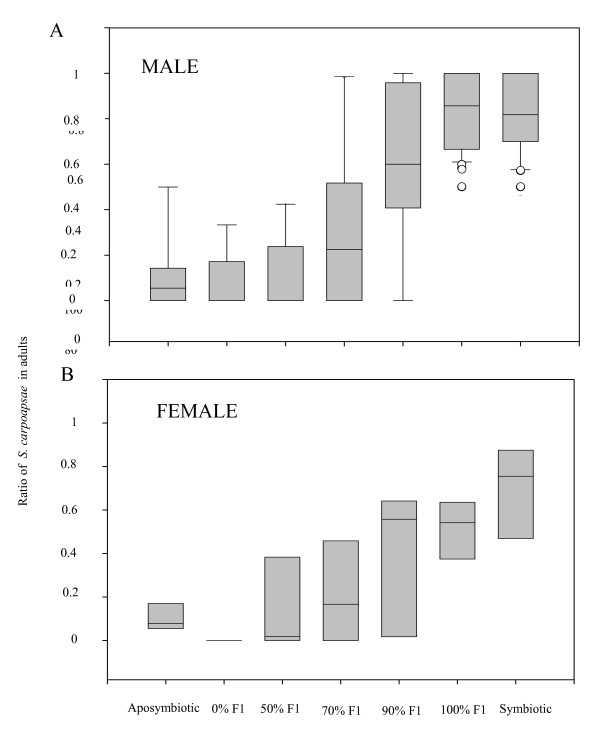
Percentile distribution of the ratio of *S. carpocapsae *within the total number of (A) nematode males and (B) nematode females found in the insect 120 h post-infection. Results are given as box plots, where the horizontal line indicate the median (50^th ^of the data), the bottom and the top of the box indicate the first quartiles (25^th ^of the data) and the third quartiles (75^th ^of the data). The whiskers the range of the data (10^th ^of the data and 90^th ^of the data). Others dots are outliers which are under the 10^th ^percentile of the data or over 90^th ^percentile of the data. The abscissa shows the condition of the initial infection.

To know if GFP labelling of *X. nematophila*, employed to discriminate the two nematode species within the global offspring, triggered differences on the progress of competition between nematodes, the data obtained with or without GFP labelling of *X. nematophila *in each competition situation were compared with a Mann Withney test. We showed that GFP labelling had no statistically significant effect on both (i) the ratio of *S. carpocapsae *among all females found in the insect 150 h after infection (N = 4; 4 <*U *< 14; 0.072 <*P *< 0.449) and (ii) the ratio of *S. carpocapsae *among all males found in the insect 150 h after infection (21 <*N *< 33; 214,000 <*U *< 398,00; 0.172 <*P *< 0.780). Because of the non-significant differences observed in these comparisons, the results obtained in each situation with and without GFP labelled *X. nematophila *were pooled in further analyses. The ratios of *S. carpocapsae *among all males found in the insects were highly heterogeneous for the different competition conditions (KW: *H *= 164.052; *P *< 0.0001). The Noether test showed that no significant difference in the ratio of *S. carpocapsae *males occurred under four competition conditions: (i) competition resulting from infection of insects with aposymbiotic IJs of both nematode species without injection of bacterium, (ii) competition resulting from infection with aposymbiotic IJs of both nematode species in insects injected with 100% of *X. innexi*, (iii) competition resulting from infection with aposymbiotic IJs of both nematode species with 50% of each symbiont injected in the insects and (iiii) competition resulting from infection with aposymbiotic nematodes in insects injected with 70% of *X. nematophila *and 30% of *X. innexi *(24 < N < 63, 0.464 <*z *< 2.568, 0.441 <*P *< 1). In these four conditions, the ratios of *S. carpocapsae *among males were significantly lower than in the three other conditions of competition: (i) competition resulting from infection with aposymbiotic IJs in insects injected with 90% of *X. nematophila *and 10% of *X. innexi*, (ii) competition resulting from infection between aposymbiotic IJs in insects injected with 100% of *X. nematophila *and (iii) competition resulting from infection with symbiotic IJs (28 < N < 63; 3.418 <*z *< 9.143; 0.0001 <*P *< 0.0264) (see Fig. 2). These results showed that an increase in the proportion of *X. nematophila *within bacteria injected into the insect triggered an increase of *S. carpocapsae *within adult worms found in the insects. The ratios of *S. carpocapsae *among females found in the insects were also highly heterogeneous for the different competition conditions (KW: *H *= 24.946; *P *< 0.001). The Noether test showed that the ratios of *S. carpocapsae *within females were lower in competition resulting from infection with aposymbiotic IJs in insects injected with 100% of *X. innexi *than (i) in competition resulting from infection with aposymbiotic IJs in insects injected with 100% of *X. nematophila *and (ii) in competition resulting from infection with symbiotic IJs (5 < N < 8; 3.260 <*z *< 4.028; 0.002 <*P *< 0.046). In competition resulting from infection with aposymbiotic IJs in insects injected with 100% of *X. innexi*, no *S. carpocapsae *females were observed.

### Assessment of the nematode offspring

#### Total number of infective juveniles (total nematode offspring)

The total number of IJs emerging from insects in the different competition situations for *S. carpocapsae *and *S. scapterisci *was heterogeneous under the different competition conditions (K.W. *H *= 71.041, *P *< 0.0001). The Noether test showed that competition resulting from infection with aposymbiotic IJs without injection of bacteria into the insect resulted in the production of significantly fewer IJs than (i) competition resulting from infection with symbiotic IJs (*z *= 7.296, *P *< 0.001), (ii) competition resulting from infection with aposymbiotic IJs in insects injected with 50% of each symbiont (*z *= 6.108, *P *< 0.0001), (iii) competition resulting from infection with aposymbiotic IJs in insects injected with 70% of *X. nematophila *and 30% of *X. innexi *(*z *= 5.174, *P *< 0.0001) and (iiii) competition resulting from infection between aposymbiotic IJs in insects injected with 90% of *X. nematophila *and 10% of *X. innexi *(*z *= 4.304, *P *< 0.001).

#### Proportion of each nematode species in the total nematode offspring

**Figure 3:**
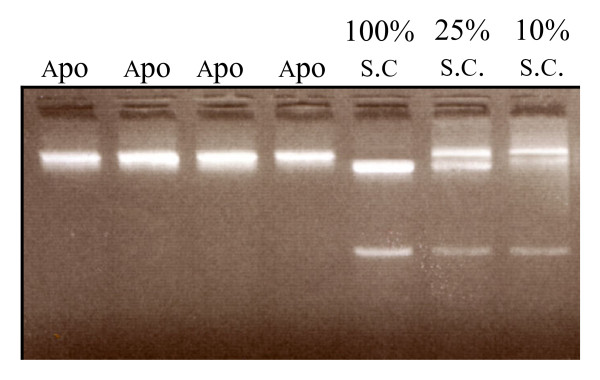
Migration of the PCR-RFLP products on agarose gel. The wells annotated (Apo) contained PCR-RFLP products coming from DNA extraction of pools of 500 IJs having emerged from competitions between aposymbiotic nematodes without the injection of bacteria. The well annotated 100% s.c contained 500 IJs of *S. carpocapsae*. The wells annotated 10% s.c and 25% s.c. respectively contained 10% of *S. carpocapsae *and 90% of *S. scapterisci *and 25% of *S. carpocapsae *and 75% of *S. scapterisci*.

**Figure 4:**
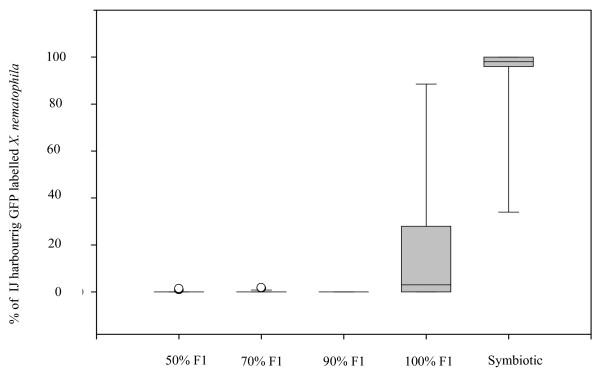
Percentile distribution of the percentages of IJs harbouring GFP labelled *X. nematophila*. Results are given as box plots, where the horizontal line indicate the median (50^th ^of the data), the bottom and the top of the box indicate the first quartiles (25^th ^of the data) and the third quartiles (75^th ^of the data). The whiskers the range of the data (10^th ^of the data and 90^th ^of the data). Others dots are outliers which are under the 10^th ^percentile of the data or over 90^th ^percentile of the data. The abscissa shows the condition of the initial infection.

In the competition situation resulting from infection with aposymbiotic IJs of both nematode species without injection of bacteria into the insect, the proportion of each nematode species in the total offspring emerging from four insects was assessed with PCR-RFLP made after global extraction of DNA from pools of 500 IJs. In control samples, the presence of 25% and 10% of *S. carpocapsae *IJs within pools of 500 IJs containing respectively 75% and 90% of *S. scapterisci *were easily detected (i.e. three bands were observed after incubation of the PCR product with Hind III) (Fig. 3). In all the four pools of 500 IJs tested here, only one band was observed after incubation of the PCR product with Hind III (Fig. 3). This result suggested that after competition between aposymbiotic nematodes without injection of bacteria, more than 90% of all IJs emerging from the insect belonged to *S. scapterisci*. In other competition situations, the proportion of each nematode species was evaluated by counting the proportion of nematodes harbouring GFP labelled *X. nematophila *within the global offspring. GFP-fluorescence of *X. nematophila *is a good indicator of *S. carpocapsae'*s IJs because pilots experiments showed that GFP labelled bacteria were still observable in IJs that were stored during 6 months at 8°C and that after several generations within different insects, at each generation, 95% of the IJs emerging from the insects habored GFP labelled bacteria. Nevertheless, in order to check that the GFP expression was not lost all along our experiments, we checked GFP expression for all the *X. nematophila *isolated from pools of 500 IJs and confirmed that none of them did not express GFP. This result confirmed the perfect stability of GFP labelled *X. nematophila *all along our experiments. Competition resulting from infection with aposymbiotic IJs in insects injected with (i) 50% of each symbiont, (ii) 70% of *X. nematophila *and 30% of *X. innexi *and (iii) 90% of *X. nematophila *and 10% of *X. innexi *gave offspring in which almost no IJs harboured GFP labelled *X. nematophila *(Fig. 4). These observations showed that *S. scapterisci *was highly dominant in the total offspring emerging from insects after such competitions. For competition resulting from infection with aposymbiotic IJs in insects injected with 100% of *X. nematophila*, the proportion of IJs harbouring GFP within the total offspring was very variable from 0 to 100% with almost all the combinations (for the distribution, see Fig. 4). For competition resulting from infection with symbiotic IJs, most of the IJs emerging from the insects (90 ± 22%) harboured GFP labelled *X. nematophila *(Fig. 4). The latter result suggested that in competition resulting from infection with symbiotic IJs, most of the offspring emerging from the insects belonged to the species *S. carpocapsae*.

## Discussion

Many invertebrates are associated with prokaryotes that provide them with the ability to live in extreme environments [15-17]. The most documented symbioses are those where symbionts provide their hosts with new abilities to take advantage of poorly nutritive resources [15,18-20]. Nevertheless, symbionts can play other important roles on diverse life history traits of their hosts, for example, the bacterium *Vibrio *which provides its sepiolids host with a better discretion towards its predators [21,22]. We already knew that *Xenorhabdus *symbionts provided *Steinernema *nematodes with diverse adaptations to their environment: (i) they produce virulence factors in order to kill the insect [23-25], (ii) they lead to a better access to the insect biomass and are themselves food for the nematodes [3,4], (iii) they produce antibiotics which counteract the multiplication of both distant and closely related bacteria [26-29]. The goal of this study was to infer the abilities of *Xenorhabdus *to modulate the progression and outcome of interspecific competition between *Steinernema *species. For this, we made experimental competitions between *S. carpocapsae *and *S. scapterisci *and their bacterial symbionts. We assessed several parameters of these competitions: (i) the proportion of each symbiont (i.e. *X. nematophila *and *X. innexi*) within the hemolymph of the insect after delay of multiplication, (ii) the ratio of *S. carpocapsae *among the adults found in the insects and (iii) the offspring of each nematode species after competition. The assessment of bacterial multiplication within insect hemolymph showed that *X. innexi *appeared more competitive than *X. nematophila*. Moreover, in insects infected with aposymbiotic IJs and in which 50% of each *Xenorhabdus *strains were initially injected, we found *X. innexi *highly dominant within the hemolymph after 48 h of multiplication (Fig. 1). Nevertheless, the ability of *X. innexi *to outcompete *X. nematophila *was not sufficient enough in the symbiotic competition where *S. scapterisci *IJs brought 700-fold less of their symbionts than *S. carpocapsae *in the insects [3]. In competition resulting from infection of insects with symbiotic IJs, our results showed that such a competition took place within the insects where only *X. nematophila *proliferated.

In this study, we studied both (i) the development of nematodes within the insects and (ii) their ability to reproduce in them. Regarding the first aspect, we showed that the nematode's development into adults differed significantly for (i) competition resulting from infection with aposymbiotic IJs of both nematode species without injection of bacteria and (ii) competition resulting from infection between symbiotic IJs of both nematode species. The competition between aposymbiotic nematodes (without injection of bacteria) allowed evaluation of the intrinsic ability of each nematode species to outcompete the other one without the help of its symbiotic *Xenorhabdus*. In such a situation, we showed that (i) significantly fewer nematodes developed into adults and that (ii) *S. scapterisci *was highly dominant in both males and females found in the insects. These results showed that *S. scapterisci *had a better competitive ability without its *Xenorhabdus *symbiont than did *S. carpocapsae *(Fig. 2). On the contrary, in the competition between symbiotic nematodes, *S. carpocapsae *was highly dominant in adults (Fig. 2). Experiments in which the amount of each *Xenorhabdus sp*. initially introduced in the insect was experimentally modified showed that the proportion of adults from each nematode species in the insects was directly linked to the proportion of their bacterial symbionts within the insect hemolymph. (Fig. 2). The more *X. nematophila *was dominant within the insect hemolymph, the more the proportion of *S. carpocapsae *in adult worms found in the insects increased. The analyses of the offspring emerging from the insects showed that competition resulting from infection with aposymbiotic IJs without injection of bacteria almost only produced IJs belonging to the species *S. scapterisci*. On the contrary, and in accordance with the results obtained on adults development, almost all the IJs emerging from the competition resulting from infection with symbiotic IJs belonged to the species *S. carpocapsae*.

Regarding the offspring production of each nematode species, the experimental modification of the bacterial symbiotic environment within the insects led to surprising results. In all insects where *X. innexi *was injected [even at the lowest dose (10% of the bacteria injected], almost no *S. carpocapsae *were found in the offspring. The inability of *S. carpocapsae *to reproduce in such situations was certainly due to the antagonistic effect of *X. innexi *toward this nematode which has already been demonstrated. Indeed, previous studies had shown that aposymbiotic *S. carpocapsae *were unable to reproduce in insects where only *X. innexi *was injected [5] but that the co-injection of *X. nematophila *with *X. innexi *led to a partially re-established reproduction of *S. carpocapsae *[13]. In the present study, even competition situations where *X. nematophila *co-infected the insects with *X. innexi *gave almost no offspring for *S. carpocapsae*. In such competition situations, it seems that *X. nematophila *was not able to counteract the effect. We can thus postulate that the intrinsic ability of *S. scapterisci *to outcompete by itself *S. carpocapsae *reinforces the antagonistic effect of *X. innexi *on *S. carpocapsae*'s reproduction. In such conditions, *S. scapterisci *and *X. innexi *acted in synergy to outcompete *S. carpocapsae *and *X. nematophila *leading to the absence of reproduction for the latter nematode. On the contrary, in the competition situation which was closest to the natural situation (i.e. symbiotic nematodes), the association *S. carpocapse*-*X. nematophila *totally outcompeted the association *S. scapterisci*-*X. innexi*. The outcome of the competition between symbiotic nematodes in favour of *S. carpocapsae *is quite understandable since this competition occurred in a bacterial environment constituted exclusively of *X. nematophila*. This dominance of *X. nematophila *within the insect hemolymph is due to the fact that *S. scapterisci *is poorly associated with its symbiont compared to *S. carpocapsae *(700-fold less cell transported) [3]. Thus, dramatically less cells of *X. innexi *than *X. nematophila *were released by the worms within the insect hemolymph.

## Conclusion

This study shows that the bacterial symbionts *Xenorhabdus *modify the progress and outcome of interspecific competitions between their *Steinernema *associated nematodes. This suggests that *Xenorhabdus *not only provides *Steinernema *with access to food sources but also furnishes new abilities to deal with biotic parameters such as competitors. Thus, the evolution of the specific association between *Steinernema *and *Xenorhabdus *in environments where interspecific competitions occurred frequently should lead to the selection for an increased number of bacterial cells retained by each nematode in order to outcompete other specific nematodes-bacteria complexes. A previous study has reported that there is dramatically different bacterial retention between some species of *Steinernema *[3]. This bacterial retention variability could be linked to differences in the biotic environments where each symbiotic association evolves. Indeed, *S. carpocapsae *is a worldwide distributed nematode contrary to *S. scapterisci *which is only found in South-America [30-32]. Moreover, *S. carpocapsae *is supposed to have a wider insect-host range than *S. scapterisci *[33]. These specific ecological traits of each *Steinernema *species suggest that *S. carpocapsae *could have evolved under a higher competition pressure than *S. scapterisci*. This evolutionary constraint could have induced a selection toward a tighter relationship (i.e. more bacterial cells by IJ) in the *S. carpocapsae*-*X. nematophila *association than in the *S. scapterisci*-*X. innexi *association.

## Methods

### Insects, nematodes and bacteria

The two *Steinernema *species naturally associated with their *Xenorhabdus *(naturally symbiotic nematodes) were established in the laboratory as soon as they were sampled by successive experimental infections of the last instar of the wax moth *Galleria mellonella*. The aposymbiotic and symbiotic IJs used for experiments were always freshly emerged from insects. Insect hosts were reared in the dark in aired plastic boxes at 28°C, 65% RH, on a diet of pollen and wax. Aposymbiotic IJs (i.e. without symbiont) of the species *S. carpocapsae *and *S. scapterisci *were obtained by disinfecting nematode eggs with a bleach solution as described previously [34]. The symbiotic IJs of *S. carpocapsae *associated with GFP (Green Fluorescent Protein) labelled *X. nematophila *(strain F1D3) were produced as described previously [34]. The GFP labeling is based on a plasmid expression which is very stable even if it is not recombined in the bacterial chromosome.

Bacterial suspensions were prepared by transferring a single colony of one bacterial strain to 5 ml of Luria-Bertani broth for liquid culture incubated at 28°C for 15 h. 100 μl of this liquid subculture was used to perform a culture to reach an optical density of 0.7 (600 nm wavelength). Before inoculation into the insect, the number of bacterial cells in each culture was counted with a Thoma cell and diluted in order to obtain a suspension of 100 cells/μl. A control of the actual number of bacteria in the injected suspension was measured by plating it onto three NBTA plates [35]. These plates were incubated at 28°C for 48 h. Then, the colonies that grew on these plates were counted. An experiment was kept only if the number of colonies ranged from 1500 to 2500.

### General settings

We performed three different competition experiments: (i) competition resulting from infection with aposymbiotic IJs without injection of bacteria into the insects, (ii) competition resulting from infection with aposymbiotic nematodes in insects followed by injection of variable proportions of their respective bacterial symbiont (*X. nematophila *and *X. innexi*) and (iii) competition resulting from infection with symbiotic IJs (i.e. IJs of nematodes naturally containing *Xenorhabdus *in their guts). In each of these competition experiments, we monitored (i) the proportion of each bacterial symbiont within the hemolymph 72 h post-infection with IJs, (ii) the proportion of males and females from each species within adult nematodes found in the insect and (iii) the proportion of each nematode species within the offspring (IJs). In order to be able to specifically distinguish IJs emerging from the cadaver, we labelled *S. carpocapsae *with its symbiotic bacteria, *X. nematophila*, hosting a plasmid expressing GFP. Previous studies showed that 96% of *S. carpocapsae *IJs were associated with *X. nematophila *and that *S. scapterisci *was unable to associate with this bacterial strain [34,36,37]. To be able to evaluate *S. carpocapsae*'s offspring within the total number of IJs emerging from an insect, we made two different sets of insects for each type of competition (except the competition between aposymbiotic nematodes without injection of bacteria): (i) one set of insects was infected with GFP labelled *X. nematophila *and (ii) one set with wild type of *X. nematophila*. (for sample sizes, see Table 1).

**Table 1:** Sample sizes for each type of experiment

Type of nematodes involved in competition	Proportion of *X. nematophila*/*X. innexi *injected into the insect	Number of insects used for the bacterial assessment	Number of insects used to assess the proportion of males of each species	Number of insects used to assess the proportion of females of each species	Number of insects used to assess the reproductive rate of each species	Total number of insects
Aposymbiotic nematodes	none	10	28	4	37	74
	0/100	10	29	5	15	54
	50/50	10*	46*	8*	33*	97
	70/30	10*	50*	8*	28*	96
	90/10	10*	52*	8*	28*	98
	100	10*	48*	8*	20*	86

Symbiotic nematodes	none	10*	63*	8*	37*	118

* half of these experiments were performed with GFP labelled *X. nematophila*

### Experimental infection

Forty IJs of each species (aposymbiotic or symbiotic depending on the type of experiment) were counted and deposited into 1.5-ml Eppendorf tubes containing a filter paper. A last instar of *G. mellonella *was then introduced into each Eppendorf, and the nematodes and the insect-host were incubated together at 24°C during 24 h. For competition resulting from infection with aposymbiotic and symbiotic IJs, no bacteria were experimentally introduced into the insect. For competition resulting from infection with aposymbiotic IJs in insects with a controlled and variable bacterial symbiont environment, 24 h after infection with aposymbiotic IJs, insects were injected with different proportions of the two bacterial symbionts (*X. nematophila *and *X. innexi*) [13]. The different proportions tested were (i) 100% of *X. innexi*, (ii) 50% of each symbiont, (iii) 70% of *X. nematophila *and 30% of *X. innexi*, (iiii) 90% of *X. nematophila *and 10% of *X. innexi *and (iiiii) 100% of *X. nematophila *(for sample sizes, see table 1).

### Proportion of each *Xenorhabdus *in insect's hemolymph 72 h post-infection with IJs

For infections made with aposymbiotic IJs followed by the injection of variable proportions of each symbiont, the proportion of each symbiont was assessed 72 h post-infection with aposymbiotic IJs (i.e. 48 h after the direct injection of *Xenorhabdus *cells into the insects). For infections made with symbiotic IJs, the same assessment was made 72 h post-infection with symbiotic IJs. To evaluate the proportion of each symbiotic bacterium within the insect, the hemolymph of each *Galleria mellonella *was collected using a syringe. The sampled hemolymph was diluted by 10^5 ^and 100 μl of the obtained suspension was plated out onto three NBTA plates. The plates were then incubated at 28°C during 48 h. After incubation, few cells from each colony were sampled with the help of sterile tooth picks and transferred in lines onto two NBTA plates containing control samples of *X. nematophila *and *X. innexi*. One of these plates was incubated at 28°C during 48 h and the other one at 37°C during 48 h. These two different incubation temperatures helped us to discriminate between the two *Xenorhabdus *strains. Indeed, both of these bacteria grew at 28°C but only *X. innexi *was also able to grow at 37°C [38]. The proportion of *X. nematophila *in the insect was then obtained by subtracting the number of colonies grown at 28°C from the number of colonies grown at 37°C and then dividing the result by the number of colonies grown at 28°C.

### Assessment of nematode maturation in competition

120 h post-infection by IJs, the insects were dissected and adult nematodes were transferred to separate Eppendorfs containing sterile Ringer. Males were then mounted with a drop of water between slide and coverslip and the species was determined after spicule observation [39]. The proportion of *S. carpocapsae *within all males found in an insect was calculated (for the number of insect analysed, see table 1). For each tested condition, all nematode females (i.e. from 2 to 37 per insect) found in four insects were separately analysed by PCR-RFLP and assigned to one nematode species. In order to do that, DNA from each female was separately extracted as described previously [40]. Then, the ITS region was amplified as described previously [39]. 10 μl of PCR products were then incubated at 37°C with 0.5 μl of the restriction enzyme Hind III, 2 μl of enzyme buffer and 7.5 μl of sterile water. Previous studies showed that Hind III cuts the amplified ITS fragment for *S. carpocapsae *and not for *S. scapterisci *[39]. After such analyses, the ratios of *S. carpocapsae *within nematode females found in each insect were calculated.

### Assessment of the nematode offspring

The total number of offspring produced by each infection was separately harvested in 50-ml Falcon flasks two months after infection and stored at 8°C. The total number of IJs produced was then evaluated under binocular microscope, using 1 ml of the suspension taken from the Falcon flask on a grid drawn on a 6-cm Petri dish. Then, for each IJ emergence coming from the competitions performed with GFP labelled *X. nematophila*, we (i) examined the vesicle of 100 IJs by epifluorescence microscopy and (ii) made isolation of bacteria contained in 500 IJs as previously described by Sicard et al., 2003. The first experiment led to assign each IJ to one nematode species. Indeed, if GFP labelled *X. nematophila *were observed within their vesicles, the IJs were assigned to *S. carpocapsae*, if not to *S. scapterisci*. The second experiment led to check if all the clones of *X. nematophila *isolated from IJs expressed GFP. In case of competition resulting from infection with aposymbiotic IJs without injection of bacteria into the insects, the presence/absence of each nematode species in the offspring was evaluated by performing PCR-RFLP (as described above) with DNA extraction on four pools of 500 IJs coming from four different insects. Preliminary experiments showed that when IJs of *S. carpocapsae *were mixed with IJs of *S. scapterisci *three bands were observed after PCR-RFLP. Such an approach led to detect 10% of IJs of *S. carpocapsae *within pools containing 90% of *S. scapterisci*. We used this latter experiment as a standard in our experiment (see Fig 3).

### Statistical analyses

In order to compare two non-parametric distributions, we used the Mann Withney test. To compare more than two non-parametric distributions, we performed a Kruskal-Wallis (KW) test followed by the pair wise comparison test of Noether [41].

## Authors' contributions

MS designed experiments, carried out some of them, interpreted the data and wrote the first draft of the manuscript. JH carried out main experiments and revised the manuscript, NLB carried out some experiments and revised the manuscript, SP helped to the design of some experiments, NB revised the manuscript and CM participated to the design of the study, the interpretation of the data and revised the manuscript. All authors read and approved the final manuscript.
